# Complex Behavior in the Dynamics of a Polymeric Biocomposite Material—“Liquid Wood”. Experimental and Theoretical Aspects

**DOI:** 10.3390/polym14010064

**Published:** 2021-12-24

**Authors:** Tudor-Cristian Petrescu, Petru Mihai, Johannes Theodorus Voordijk, Valentin Nedeff, Dorin Văideanu, Florin Nedeff, Traian-Dănuț Babor, Decebal Vasincu, Maricel Agop

**Affiliations:** 1 Department of Structural Mechanics, Faculty of Civil Engineering and Building Services, “Gheorghe Asachi” Technical University of Iași, 700050 Jassy, Romania; tudor.petrescu@tuiasi.ro; 2 Department of Concrete Structures, Building Materials, Technology and Management, Faculty of Civil Engineering and Building Services, “Gheorghe Asachi” Technical University of Iași, 700050 Jassy, Romania; petru.mihai@academic.tuiasi.ro (P.M.); traian-danut.babor@academic.tuiasi.ro (T.-D.B.); 3 Department of Civil Engineering, Faculty of Engineering Technology, University of Twente, 7552 LW Enschede, The Netherlands; j.t.voordijk@utwente.nl; 4 Department of Industrial Systems Engineering and Management, Faculty of Engineering, “Vasile Alecsandri” University of Bacău, 600115 Bacau, Romania; vnedeff@ub.ro; 5 Department of Natural Sciences and Mathematics, Faculty of Physics, “Alexandru Ioan Cuza” University of Iași, 700506 Jassy, Romania; naturaone@gmail.com; 6 Department of Environmental Engineering and Mechanical Engineering, Faculty of Engineering, “Vasile Alecsandri” University of Bacău, 600115 Bacau, Romania; florin_nedeff@ub.ro; 7 Department of Biophysics and Medical Physics, Faculty of Medicine, “Grigore T. Popa” University of Medicine and Pharmacy, 700115 Jassy, Romania; decebal.vasincu@umfiasi.ro; 8 Department of Physics, Faculty of Machine Manufacturing and Industrial Management, “Gheorghe Asachi” Technical University of Iași, 700050 Jassy, Romania; 9 Academy of Romanian Scientists, 050094 Bucharest, Romania

**Keywords:** liquid wood, biocomposites, tension, compression, multifractal, constitutive laws, operational procedures

## Abstract

The purpose of the present paper is to analyze, both experimentally and theoretically, the behavior of the polymeric biocomposite generically known as “liquid wood”, trademarked as Arbofill. The experimental part refers to the mechanical performance in tension and compression, having as finality the possibility of using “liquid wood” as a material suitable for the rehabilitation of degraded wooden elements in civil structures (ex. use in historical buildings, monuments etc.). The theoretical part refers to computer simulations regarding the mechanical behavior of “liquid wood” as well as to a theoretical model in the paradigm of motion, which describes the same behavior. This model is based on the hypothesis that “liquid wood” can be assimilated, both structurally and functionally, to a multifractal object, situation in which its entities are described through continuous, non-differentiable curves. Then, descriptions of the behavior of “liquid wood”, both in the Schrödinger-type and in hydrodynamic-type representations at various scale resolutions, become operational. Since in the hydrodynamic-type representation, the constitutive law of “liquid wood” can be highlighted, several operational procedures (Ricatti-type gauge, differential geometry in absolute space etc.) will allow correlations between the present proposed model and the experimental data. The obtained results, both practical (81% bearing capacity in compression and 36% bearing capacity in tension, compared to control samples) and theoretical (validation of material performance in virtual environment simulations, stresses and strains correlations in a theoretical model) indicate that “liquid wood” could be used in the construction industry, as a potential rehabilitation material, but with more development clearly needed.

## 1. Introduction

The construction industry is regarded as a major contributor to environmental pollution. It is mainly fossil-fuel driven (especially referring to product manufacturing processes) and construction sites represent a source of noise and particulate pollution. In particular, the released contaminants (such as nitrous oxides, carbon monoxides, volatile organic compounds-VOC and fine particulate matter-PM10) can travel long distances and are harmful both for humans and the environment [1]. Adding to these issues is the fact that the construction industry does not make use of sustainable and environmentally friendly products, with the notable exception of timber and stone (to some extent) [2].

Thus, one way to mitigate pollution is to use new, environmentally compliant construction materials which show promise in tackling the pollution issue. Ideally, these materials should be naturally sourced and biodegradable, at least to a certain extent [3].

One way to achieve this is to study and use biocomposite materials. A biocomposite can be defined as a composite material made out of a matrix (preferably natural, can be biodegradable) reinforced with natural fibers.

Biocomposites, although known for several decades, are regarded as being “young” [4] compared to classical building materials used for centuries, such as natural stone, timber, bricks or concrete. There are many applications of biocomposite materials, in a wide range of domains [5]. Their advantages are well known, such as good mechanical, acoustic, and thermal properties, along with flexibility in use and having a reduced environmental impact [6]. However, they have disadvantages as well, chiefly increased costs, which can spawn other negative effects, such as lower quality biocomposite products (in order to remain competitive on the market) [7].

Unfortunately, when referring to biocomposites in the context of the construction industry, their adoption and use is lagging behind, in contrast to other economic branches. In particular, structural applications, which are essential for the successful employment of biocomposites in the construction industry, are sorely lacking [8,9]. It can be argued that, with the notable exception of a few structurally aimed projects [10,11], the accelerated development of such applications is necessary. As previously mentioned, costs are an important factor when taking into account the possible replacement of “classic” building materials (concrete, stone, bricks) with sustainable ones. Concrete use, for example, is ubiquitous, so a potential competitor will have to exhibit similar “economic characteristics”, such as low production costs, flexibility in use and well-understood properties.

As a remark, it should be noted that biocomposites represent an entire class of products, with uses in several fields. Tannins, for example, are natural phenolic compounds which have many uses in medical and pharmacological industries, but they are also present in the beverage and clothing industry [12]. Another example of a promising product derived from the processing of lignin are lignosulfonate adhesives. They have shown promise in replacing industrial formaldehyde-based adhesives in the wood processing industry and may become a mainstay product in timber-related industries and industrial processes [13]. Furthermore, they do not appear to have any negative effects on animals and humans [14].Yet another way of employing lignin is by means of hydrolysis lignin, which can be used in the production of fiberboards [15]. The hydrolysis process can also be used to obtain HPH (high pressure hydrolysis)-lignin with natural fibers in the form of injectable granules [16], in a presentation manner very similar to how injectable thermoplastics are commercially available. Even more, chitosan (a polysaccharide) seems to have good adhesive properties as well, again when being used in the context of wood processing [17], but it can also have medical uses [18].

Many other applications are currently studied and much work has been devoted to the application of tannins. Especially when it comes to adhesives, a pressing issue is represented by the need to eliminate toxic formaldehydes from wood adhesive products. Not just tannin, but also urea adhesives, protein adhesives, carbohydrate adhesives, and furanic resins, as well as unsaturated oil adhesives represent promising alternatives. Natural high tech laminates and impregnated paper laminates—also employing tannins—have the potential to deliver alternative products to the industry, with good performance. Bio-based resins, foams and preservatives are also of interest for various applications [19]. Moreover, tannin matrix and natural fiber unidirectional biocomposites have been studied, being obtained in the form of woven mats. They could be interesting developments with possible applications in the field of civil engineering [20]. Additionally, bio-based resins, also derived from tannin, are interesting for the automotive and construction industries, along with use in electrical, electronic, consumer and industrial—grade goods [21]. It is possible to obtain environmentally friendly wood particleboard panels prepared with equally environmentally friendly tannin adhesives, which again can be a good alternative to industrial formaldehydes [22,23]. Regarding lignin-based adhesives and lignosulphonates, their use is also investigated. They seem to be a good candidate in replacing industrial products in the manufacture of wood panels, but more research is needed regarding their properties, interactions with other additives, and bonding mechanisms [24,25,26,27,28,29]. Adhesives containing starch, soy protein, and chitosan are also investigated, with promising outlooks and future prospects [30]. Finally, employing chitosan adhesives in the obtainment of wood panels yielded promising results regarding mechanical strengths, with the possibility of upscaling for industrial use [31,32].

All in all, these natural and naturally derived products have the potential to replace “classic”, industrially made, and oftentimes polluting products and associated processes. They are not without issues (for example, when it comes to their processing and employment), but they hold the promise of reducing the environmental impact of the industries in which they may be used.

Because the range of biocomposites is relatively large, this article will only refer to a specific polymeric biocomposite material which has shown promise for use in the construction industry—“liquid wood”.

As such, the aim of this paper is to explore, both theoretically and practically, the possible use of “liquid wood” as a structural rehabilitation material in the construction industry, in order to facilitate the development and eventual deployment of such materials.

Thus, in the present paper, both experimentally and theoretically, the behavior of the polymeric biocomposite generically known as “liquid wood”, trademarked as Arbofill, is analyzed.

A biocomposite is a type of material that exhibits a structure similar to the ones found in other types of composites, such as fiber-reinforced polymers, fiberglass etc., but offers biocompatibility. Just like in the case of “classic” composite materials, they can have the role of taking over various efforts that the material is subjected to. They tend to have relatively low density (for example, when compared to reinforced concrete or steel) and a lightweight structure, which offer traction resistance and insulation against heat and noise. Their method of weaving (as the fibers can be woven in different patterns, for example in the case of 3D-composites) can be of influence to the overall characteristics of the biocomposite [16].

As a classification, biocomposites are classified according to the type of fibers: wooden and non-wooden fibers.

Because wooden fibers are, in one way or another, part of the (global) wooden mass, they are less popular in the industry (again, compared to more traditional building materials) [33].

According to previous statements, “liquid wood” can thus be described as a polymeric biocomposite material, having a matrix made of lignin and other constituents (but mostly organic fibers). From a material constituency point of view, “liquid wood” can be thus viewed as representative for biocomposite products. Obviously, product data sheets exist for all three forms of presentation, which give information about method of processing, method of delivery, and some basic material data (for additional information, please see the “Acknowledgements” section). Each of these presentation forms contains varying proportions of lignin, but all of them are biodegradable (up to 100%) and are environmentally friendly [9,34,35].

“Liquid wood” can be processed much like existing thermoplastics, using well-known manufacturing techniques (such as hot drawing, extrusion or injection molding), hence its “polymeric” trait. While this very trait makes “liquid wood” somewhat different than other composites and biocomposites, it may be viewed as an advantage in lieu of the fact that, for certain types of applications, various objects can be obtained by making use of existing injection machines.

As an additional advantage, “liquid wood” can be reused several times over and thus processed and reprocessed, without any loss in its properties [36].

**Figure 1:**
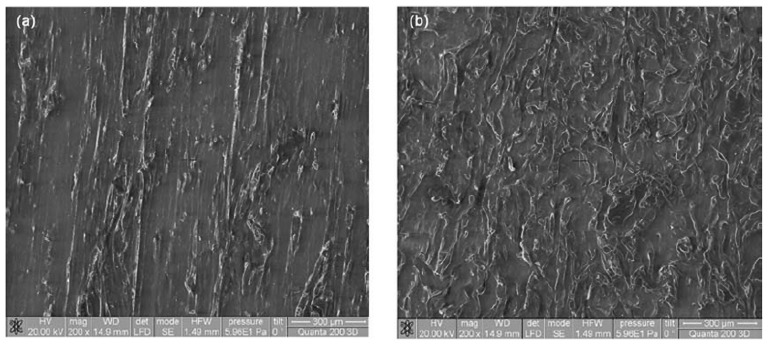
SEM images of “liquid wood”: (**a**) initial sample, (**b**) fractured sample [49].

A multitude of tests and observations have been carried out so far on “liquid wood”. These tests approached the topics of: biodegradability [37], the influence of the reinforcing material [34,38], tensile strength [39,40], microindentation [41,42], bending resistance [43,44], impact resistance [45], calorimetry [42], thermal properties [46], electrical properties [46], effect of UV light [47], surface analysis [48], and SEM analysis (several results can be visualized below, in Figure 1a,b) [49].

**Figure 2:**
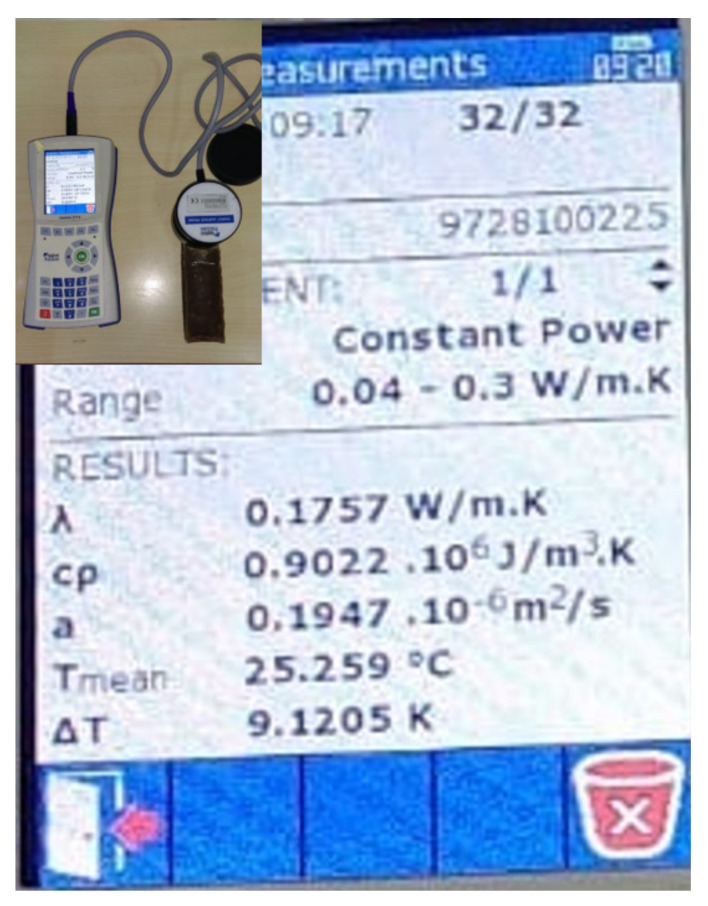
Isomet 2114 Portable Heat Transfer Analyzer next to results obtained from the Arbofill sample (screen capture) [55].

**Figure 3:**
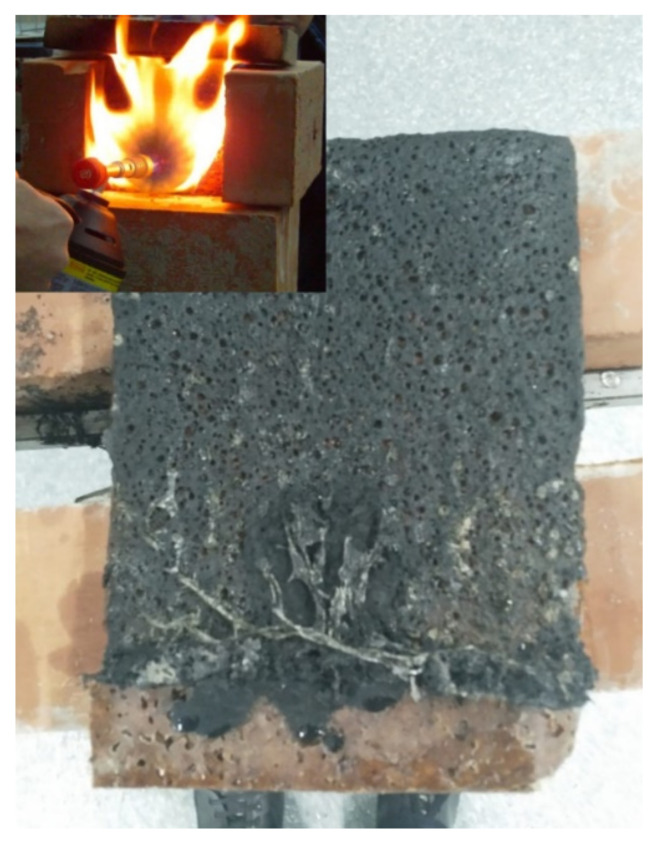
“Liquid wood” fire testing and “peeling effect” of burnt layers [55].

Because the previously mentioned tests were carried out on one, several, or all of the “liquid wood” presentation forms, some of the authors of the present paper carried out further research where it was deemed necessary to obtain data for all three material presentation forms. It should be noted that said research had, as a purpose, the study and uncovering of material properties which are relevant for the possible use of “liquid wood” in civil engineering. These properties represent a departure from the common material properties which are usually encountered in scientific literature (such as, for example, impact resistance, thermal properties, surface analysis etc., mentioned above). They are listed and briefly discussed below:

(a) Marine environment degradation—Because “liquid wood” is a up to 100% biodegradable natural material, it is necessary to study its biodegradability and the conditions in which this will occur. Thus, a study was performed on all three presentation forms when they are subjected to the effects of seawater and the microorganisms found in it. It was discovered that “liquid wood” is selective with respect to the absorbed substances and the biofilm developed on the surfaces of samples depends on the surface characteristics. As such, structural degradation is likely to occur and, if “liquid wood” is to be employed in such environments, protection (for example with bactericidal coatings) is recommended [50].
(b) Acidic/Base/UV testing—A rather comprehensive testing of all three presentation forms of “liquid wood”, immersed in water at different temperatures and in the presence of acidic, base and UV environments was carried out. This again had the purpose of determining the behavior of “liquid wood” when subjected to degradation factors. Again, the samples from all three presentation forms showed degradation, so again care must be taken with the conditions in which “liquid wood” is employed [51].
(c) Thermal properties—Because a study of thermal properties was made only on the “liquid wood” trademarked as Arbofill, the other trademarked products—Arboblend and Arboform—were analyzed as well, concerning calorimetric aspects as well as XRD and FTIR procedures. These tests help to further optimize the material injection process [52].
(d) Electrical properties—The scenario mentioned above was repeated for the study of electrical properties. As such, it resulted that both Arboform and Arboblend trademarked commercial products can be considered as electric insulator materials [52].
(e) Biostructure dynamics—It is possible to look at polymeric biocomposite materials as biostructures (since they contain lignin, as is the case for “liquid wood”, or other natural and naturally—derived materials). Then, it is possible to view such biostructures in terms of fractals and multifractals. Finally, both time and critical velocity particular to any biostructure can be defined (as in biological time and growth speed). Translated to an engineering point of view, this type of analysis can describe the growth of plant material and thus be used as an estimate of material production (for example, lignin) per year [53].
(f) Thermal insulation properties—For a material which has the potential to be used in the construction industry, if appropriate, its thermal insulation properties are usually studied. If they are satisfactory, then said material may be technically considered as a thermal insulator. On a trademarked Arbofill specimen of dimensions 155 × 35 × 35 mm, a reading was made using an Isomet 2114 measuring device [54] (see Figure 2), with an internal porous structure (resulting as a consequence of the sample obtainment method). The retrieved data (see Figure 2) showed an insulating performance similar to low/medium performance ceramic bricks [55].
(g) Fire behavior—Any material that is slated to be used in a building should be tested to the action of fire. A trademarked Arbofill product plate of dimensions 70 × 120 × 10 mm was tested in the scenario of being used as wall cladding, with a violent fire (800–1200 °C) starting next to it (see Figure 3). The “liquid wood” did not exhibit any noteworthy fire resistance, but it does have one desirable property—it burns in a very gradual manner, melting away layer by layer and then flowing (see Figure 3) [55]. Since there are no currently standing testing standards for fire behavior of biocomposite materials, the test method was also scenario-based.
(h) Rheological properties—Rheology is concerned with the study of flow of solids, but also of non-Newtonian fluids. All “liquid wood” presentation forms were subjected to such testing. The concluding results allow for the determination of optimal viscosity of these materials, in the context of thermal injection [56].

These tests collectively seem to point out that this material could be used in a structural role in various applications pertaining to the construction industry. Out of all three presentation forms, Arbofill trademark product seems to be the one that has the most suitable properties for use in the construction material. Nevertheless, so far there has been little attempt to employ “liquid wood” in a structurally appropriate manner, much of the research still being relegated to a laboratory environment.

## 2. Experimental Aspects

In order to assess the suitability of the “liquid wood” Arbofill trademark product for use in building rehabilitation, a scenario was proposed, on which the experiment should rely, in order to replicate an on-site rehabilitation procedure. The idea was that, since “liquid wood” can be thermo-injected, it could be used as a filler material for degraded wooden elements (ex. beams, columns), by cutting out the damaged part of said wooden element. Then, by filling the resulting groove with “liquid wood”, the bearing capacity of the respective beam or column, for example, could be restored. This scenario was replicated in the research team’s laboratory. As a point of note, regarding the (eventual) use of “liquid wood” in the construction industry, the material should be accompanied by certificates of approved testing institutes. The experiment presented below only addresses a very specific scenario related to constructions.

It is very important to underline the fact that the aim of the experiment is not to study the characteristics of “liquid wood”, but rather the behavior of the timber elements retrofitted with “liquid wood”, in order to determine its viability for use as a repair material.

As such, a collection of 12 wooden beams of wood grade class C16 were prepared, in accordance to EN 14081-1. They have the dimensions of 20 × 50 × 800 mm, as recommended by EN 408-2004, and were made ready for testing, by creating a 2 × 2 cm or 2 × 4 cm groove in the mid-section, with the help of a power saw. Nine beams were processed as such, with the remaining three being kept as witness (unmodified) samples. It is acknowledged that this is the absolute minimum of samples that can be tested in order to get appropriate results, according to EN 408-2004. It was decided to use this number of samples as, in previous research, the authors found out that the obtained results are consistent and therefore it would not make a lot of sense to prepare and process a large amount of test beams [55].

Out of the resulting nine beams, six of them, made with the 2 × 2 cm groove in the mid-section, were tested in both tension and compression by bending, in batches of three. The remaining three beams, made with the 2 × 4 cm groove in the mid-section, were tested in tension by bending.

**Figure 4:**
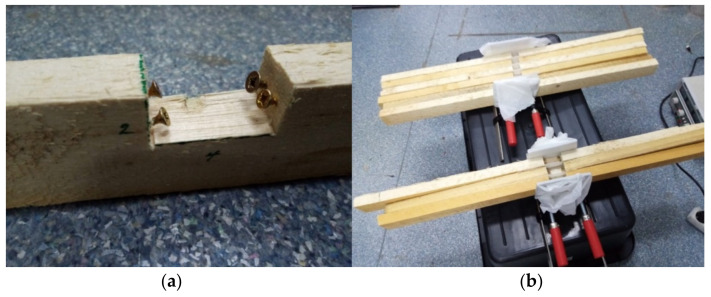
Wooden beams used in bending test: (**a**) wood screws installed in 2 × 4 cm groove beam; (**b**) 2 × 2 cm groove beams and 2 × 4 cm groove beams clamped in separate stacks.

Furthermore, in an attempt to improve the adhesion properties of “liquid wood” to regular timber, it was decided to fit the three beams that had a 2 × 4 cm groove with wood screws (see Figure 4a), in various configurations.

After all preparations were done, the beams were thermo-injected with the product in the grooves. The injection procedure was carried out with the help of a portable thermo-injection machine, which is currently the subject of a pending patent request, No. A/00572/2020, registered with the Romanian State Office for Inventions and Trademarks. The used material volume is estimated at approximately 100 cm^3^ of granules.

The beams were clamped together in two separate stacks—one containing the beams with 2 × 2 cm groove and one containing the beams with 2 × 4 cm groove, in order to facilitate the injection, which is a continuous working process. The grooves were separated with common baking paper, which has the role of a non-stick medium for “liquid wood”. The baking paper was level cut with the beams, in order to not interfere with the continuity of the injection process (see Figure 4b).

An attempt was made to remove the excess “liquid wood”, however this proved unsuccessful, as the material rapidly cools after injection and becomes quite hard. Manual-based attempts to remove the excess material were unsuccessful, but it can be theorized that mechanical grinding may achieve the desired results.

**Figure 5:**
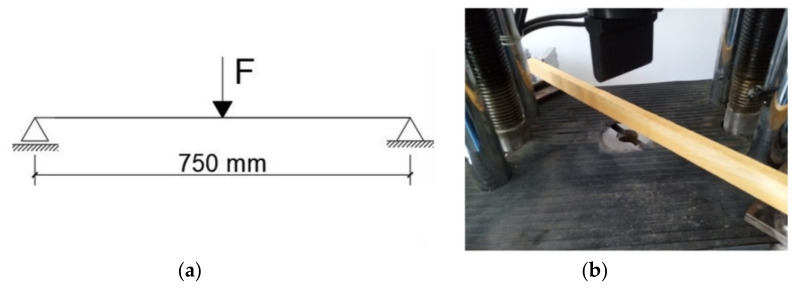
Laboratory three-point bending test setup: (**a**) static scheme of three-point bending test, (**b**) test setup on plain wooden beam.

After being left to cool and settle for approximately two days, the two beam stacks were unclamped and arranged for structural testing. In order to achieve this, a 600 kN testing machine, model WDW 50 with proprietary acquisition software, was used. The three-point bending test, as previously mentioned, was carried out, with a distance between the supports of 750 mm (see Figure 5a).

The testing procedure is fairly straightforward. The beam is settled on supports located at each end of the wooden element. It is then carefully aligned with the loading cell, which applies the force in the middle of the beam, in increments of 10 N/s, until failure. The sensors and the software of the press record the results. Specifically, two parameters are recorded: applied force and resulting displacement.

**Figure 6:**
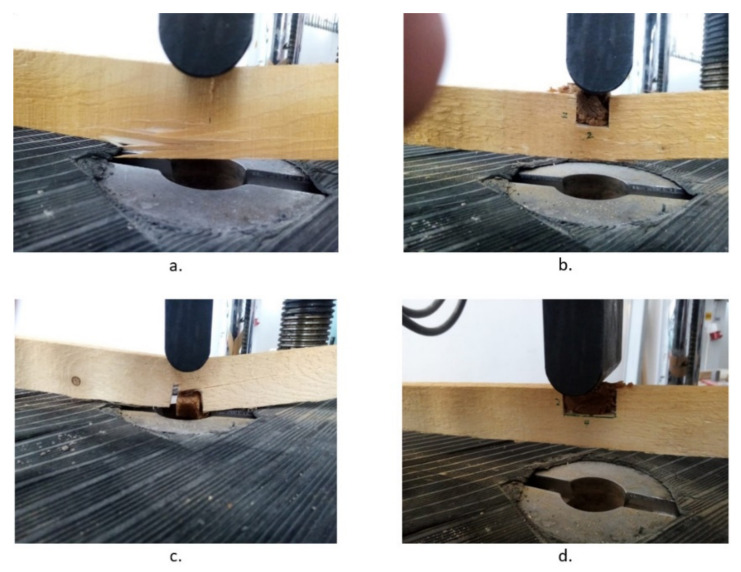
Failure modes of plain and “liquid wood” retrofitted wooden beams: (**a**) bending failure of the plain wooden beam; (**b**) failure in bending by compression of retrofitted 2 × 2 groove beam; (**c**) failure in bending by tension of retrofitted 2 × 4 groove beam; (**d**) cell load and deflection in bending by compression of retrofitted 2 × 4 groove beam.

First, the plain wooden beams were tested, in order to extract the control/witness values, which were very close to the standard stipulated ones. They represent the benchmark against which the performance of the rehabilitated wooden elements is evaluated (see Figure 5b and Figure 6a).

Second, the wooden beams with the 2 × 2 groove were tested in tension and compression by three-point bending (see Figure 6b,c). The same procedure previously described was followed.

Third, the wooden beams with the 2 × 4 groove were tested in tension and compression by three-point bending (see Figure 6d). The same procedure previously described was followed.

**Figure 7:**
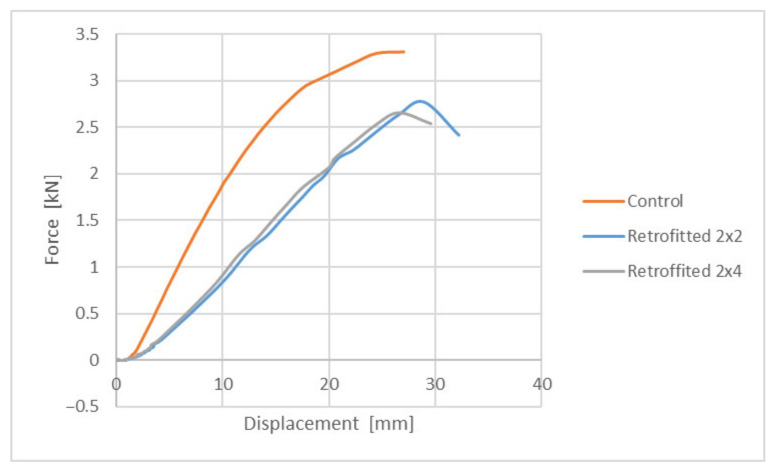
Behavior of samples rehabilitated with “liquid wood” subjected to compression.

**Figure 8:**
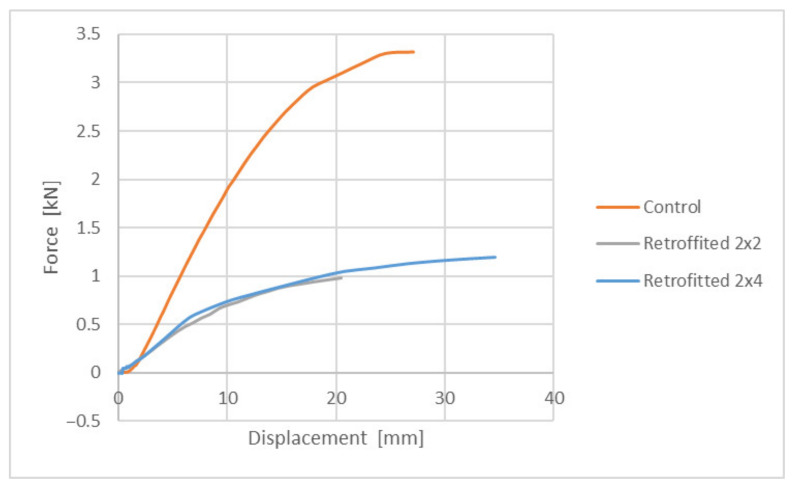
Behavior of samples rehabilitated with “liquid wood” subjected to tension.

The overall results are summarized, as an average of values, in the following graphs (see Figure 7 and Figure 8).

All the tests went according to the initial expectations, with no brittle or unanticipated failures occurring.

## 3. Theoretical Aspects

### 3.1. Computer Simulations

Almost any material may be studied in a virtual environment. If the material characteristics are known, then they can represent input data in specific simulation software. As such, the studied material can be subjected to various loads, loads that would occur in a projected use, in a specific field. The results obtained following computer simulations can provide important data, for example related to the reliability and performance of a product.

In the case of “liquid wood”, it was decided to use Autodesk Inventor virtual simulation environment, in line with the expected employment of “liquid wood” as a thermo—injectable material into existing elements. The simulation software is capable of rendering and analyzing “solids” (3D objects), even when distinct materials are involved (which is in line with the idea of using “liquid wood” as a solution for the repairing of wooden elements).

Simulations were made of wooden beams which were rehabilitated with “liquid wood”, in a very similar scenario/setup to the one presented in the practical application.

The product was tested in three-point bending at a force of 10 kN. The lateral supports/restrains and the acting force were defined.

**Figure 9:**
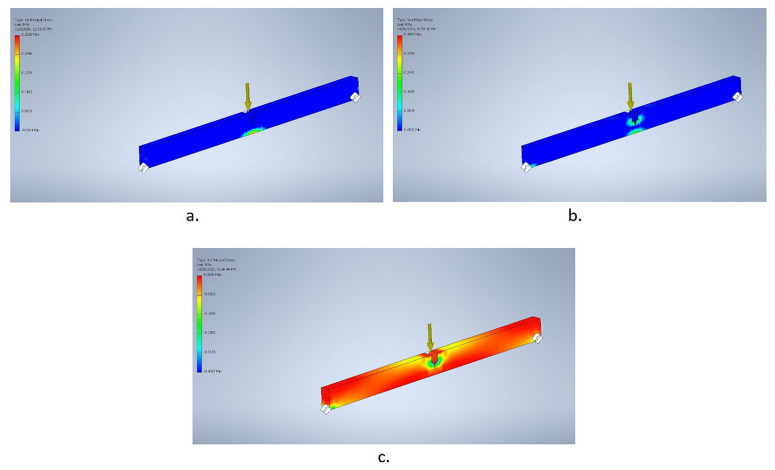
Results from the computer simulation on product sample: (**a**) 1st principal stresses; (**b**) Von Misses stresses; (**c**) 3rd principal stresses.

Then, by “running” the simulation, the resulting output of data were displayed. Furthermore, a colored effort map and an associated value scale were also present (see Figure 9a–c).

The obtained values are similar to the experimental ones. This aspect is of particular importance, as it confirms the correspondence of the virtual simulation to the “real” results obtained in the laboratory (hence, it confirms the performance of the rehabilitation solution). Such types of modeling and numerical simulations are recommended, as they are cheaper and faster than carrying out laboratory experiments. They have the potential to provide useful data, as long as the input values reflect expected loads to be encountered in actual product use.

### 3.2. Mathematical Model

#### 3.2.1. General Considerations on the Multifractal Theory of Motion

Common models used to describe the dynamics of biocomposites, are based either on a combination of basic theories, derived especially from physics or on computer simulations [57,58,59,60,61,62]. In such a context, their description implies both computational simulations based on specific algorithms [60], as well as developments on standard theories. With respect to models developed on standard theories, the following classes can be distinguished:

(i) A class of models developed on spaces with integer dimension—i.e., differentiable models (for example, developments based on Generalized Hooke Laws etc.) [57,58].
(ii) Another class of models developed on spaces with non–integer dimensions, which is explicitly written through fractional derivatives [61,62]—i.e., non-differentiable models, with examples including the fractal models [63] (It is reminded that a fractal represents a subset of Euclidean space, having a fractal dimension which strictly exceeds its topological dimension. A multifractal represents a generalization of the fractal, in the sense that it is a set of fractals. For details see [63,64]).
(iii) Expanding the previous class of models, new developments have been made, based on Scale Relativity Theory, either in the monofractal dynamics as in the case of Nottale [63], or in the multifractal dynamics as in the case of the Multifractal Theory of Motion [64].

The fractal approach to studying the behavior of “liquid wood” when used in conjecture with regular timber is favorable, as the material exhibits a complex structure. Both in the context of Scale Relativity Theory in the sense of Nottale [63], as well as in the one of Multifractal Theory of Motion [64], the fundamental hypothesis is the following: any type of biocomposite (for example, in the present context, “liquid wood” in its various trademarked forms) is assimilated both structurally and functionally to a multifractal object (for example, see Figure 1a,b, which show self-structuring modes of “liquid wood”). From such a perspective, the dynamics of the polymeric biocomposites can be described through motions on continuous and non-differentiable curves (multifractal curves), of the structural units belonging to the polymeric biocomposite material (assimilating the polymeric biocomposite with a complex system, it is possible to operate in its dynamics with structural units).

Such a hypothesis may be clarified by considering the following scenario: between two successive interactions of the structural units belonging to any polymeric biocomposite, the trajectory of the structural unit belonging to the polymeric biocomposite is a straight line that becomes non-differentiable in the impact point. Considering that all interaction points form an uncountable set of points, it results that the trajectories of the structural units belonging to the composite become continuous and non-differentiable (i.e., fractal curves). Evidently, the reality is much more complicated, taking into account both the diversity of the structural units which compose any polymeric biocomposite and the various interactions between them in the form of double interactions etc. Extrapolating the previous reasoning for any type of polymeric biocomposite, including the “liquid wood” in its various presentation forms, it results that it can be assimilated to a multifractal.

All these considerations imply that, in the description of the “liquid wood” dynamics in its various presentation forms, instead of “working” with a single variable (regardless of its nature, i.e., velocity, density, etc.) described by a strict non-differentiable function, it is possible to “work” only with approximations of this mathematical function, obtained by averaging them on different scale resolutions. As a consequence, any variable purposed to describe “liquid wood” dynamics in its various presentation forms will perform as the limit of a family of mathematical functions, this being non-differentiable for null scale resolutions and differentiable otherwise [63]. Otherwise said, from a mathematical point of view, these variables can be explained through multifractal functions (i.e., functions dependent not only on spatial and temporal coordinates, but also on the scale resolution).

Since for a large temporal scale resolution with respect to the inverse of the highest Lyapunov exponent [65,66], the deterministic trajectories of any structural unit belonging to the “liquid wood” in its various presentation forms, can be replaced by a collection of potential (“virtual”) trajectories. Then, the concept of definite trajectory in the description of “liquid wood” dynamics in its various presentation forms, can be substituted by the one of probability density.

Taking into account all of the above, the multifractality expressed through stochasticity, in the description of “liquid wood” dynamics in its various presentation forms, becomes operational in the multifractal paradigm through the Multifractal Theory of Motion. Various applications of this model are given in [65,66,67,68,69,70,71,72,73].

The present analysis was directed to the modeling of the behavior of “liquid wood”, more precisely, the tension and compression phenomena. A mathematical model was created considering “liquid wood” as a multifractal object, and analyzing its dynamics in the framework of Scale Relativity Theory, in the form of hydrodynamic descriptions, using various operational procedures (Riccati-type gauges and invariance groups). Finally, the theoretical model was validated by means of the experimental data.

#### 3.2.2. Consequences of Non-Differentiability in a Multifractal Paradigm

Considering “liquid wood” as a multifractal object, in what follows, it is admitted that the motions of the entities of any polymeric biocomposite are described by continuous and non-differentiable curves (multifractal curves).

Then, the motion equation of the entities of any polymeric biocomposite becomes (for details see [64]):

(1) $$\frac{\hat{d}{\hat{V}}^{i}}{dt}=\partial_{t}{\hat{V}}^{i}+{\hat{V}}^{l}\partial_{l}{\hat{V}}^{i}+\frac{1}{4}{\left(dt\right)}^{\left[\frac{2}{f\left(\alpha \right)}\right]-1}D^{lk}\partial_{l}\partial_{k}{\hat{V}}^{i}=0.$$

where

(2) $${\hat{V}}^{l}=V_{D}^{l}-V_{F}^{l}\;D^{lk}=d^{lk}-i{\hat{d}}^{lk}\;d^{lk}=\lambda_{+}^{l}\lambda_{+}^{k}-\lambda_{-}^{l}\lambda_{-}^{k}\;{\hat{d}}^{lk}=\lambda_{+}^{l}\lambda_{+}^{k}+\lambda_{-}^{l}\lambda_{-}^{k}\;\partial_{t}=\frac{\partial }{\partial t},\;\partial_{l}=\frac{\partial }{\partial x^{l}},\;\partial_{l}\partial_{k}=\frac{\partial }{\partial x^{l}}\frac{\partial }{\partial x^{k}},\;i=\sqrt{-1},\;l,k=1,2,3$$

In (2), the meaning of the variables and parameters are as follows:

- $x^{l}$ is the multifractal spatial coordinate;
- t is the non-multifractal time having the role of an affine parameter of the motion curves;
- ${\hat{V}}^{l}$ is the multifractal complex velocity;
- $V_{D}^{l}$ is the differentiable velocity independent on the scale resolution;
- $V_{F}^{l}$ is the non-differentiable velocity dependent on the scale resolution;
- dt is the scale resolution;
- $f\left(\alpha \right)$ is the singularity spectrum of order α;
- α is the singularity index and is a function of fractal dimension $\;D_{f}$;
- $D^{lk}$ is the constant tensor associated with the differentiable–non-differentiable transition;
- $\lambda_{+}^{l}\left(\lambda_{+}^{k}\right)$ is the constant vector associated with the backward differentiable–non-differentiable dynamic processes;
- $\lambda_{-}^{l}\left(\lambda_{-}^{k}\right)$ is the constant vector associated with the forward differentiable–non-differentiable dynamic processes;

By using the singularity spectrum, the following patterns in the polymeric biocomposite dynamics can be distinguished: monofractal patterns which imply dynamics in homogenous polymeric biocomposites characterized though a single fractal dimension and having the same scaling properties in any time interval; multifractal patterns that include dynamics in inhomogeneous and anisotropic polymeric biocomposites characterize simultaneously by a wide variety of fractal dimensions. Thus *f(α)* allows the identification of the universality classes in the dynamics of any polymeric biocomposite even when the strange attractors associated to these dynamics have different aspects. For details on the singularity spectrum and its implication on the dynamics of complex systems see [74,75,76].

For a large temporal-scale resolution with respect to the inverse of the highest Lyapunov exponent [74,75], the class of deterministic trajectories of any polymeric biocomposite entities can be substituted by the class of virtual trajectories. Then, the concept of definite trajectories is replaced by the one of density of probability. The multifractality is then expressed by means of multi-stochasticity and becomes functional when describing the dynamic of any polymeric biocomposite in the form of multifractal fluid dynamics (for details see [64]).

Many modes of multifractalization through stochasticization processes can be defined. Among the most utilized processes, the Markovian and non-Markovian stochastic processes are found [75,76]. In what follows, in the description of polymeric biocomposite dynamics, only multifractalizations by means of Markovian stochastic processes will be discussed, i.e., those specified by constraints [75,76]:

(3) $$\lambda_{+}^{i}\lambda_{+}^{l}=\lambda_{-}^{i}\lambda_{-}^{l}=2\lambda \delta^{il}$$

where λ is a constant associated to the differentiable-nondifferentiable transitions and $\delta^{\mathrm{il}}$ is the Kronecker pseudo-tensor. Based on (3), the motion Equation (1) become (for details on the mathematical procedure see [64]):

(4) $$\frac{\hat{d}{\hat{V}}^{i}}{dt}=\partial_{t}{\hat{V}}^{i}+{\hat{V}}^{l}\partial_{l}{\hat{V}}^{i}-i\lambda {\left(dt\right)}^{\left[\frac{2}{f\left(\alpha \right)}\right]-1}\partial_{l}\partial^{l}{\hat{V}}^{i}=0.$$

The relations (4) show that, in any point of the motion curves, the local multifractal complex acceleration, $\partial_{t}{\hat{V}}^{i}$, the multifractal complex convection, ${\hat{V}}^{l}\partial_{l}{\hat{V}}^{i}$, and the multifractal complex dissipation $i\lambda {\left(dt\right)}^{\left[\frac{2}{f\left(\alpha \right)}\right]-1}\partial_{l}\partial^{l}{\hat{V}}^{i}$ are in equilibrium.

In what follows, let it be admitted that the motions of the entities belonging to any polymeric biocomposite are irrotational. Then, the multifractal complex velocity fields from (2) become:

(5) $${\hat{V}}^{i}=-2i\lambda {\left(dt\right)}^{\left[\frac{2}{f\left(\alpha \right)}\right]-1}\partial^{i}\ln\Psi$$

where

(6) $$\chi =-2i\lambda {\left(dt\right)}^{\left[\frac{2}{f\left(\alpha \right)}\right]-1}\ln\Psi$$

is the multifractal complex scalar potential of the complex velocity fields from (5) and Ψ is the function of states (on the significances of Ψ, see [74,75]). In these conditions, substituting (5) in (4) and using the mathematical procedures from [74,75] the motion Equation (4) takes the form of the multifractal Schrödinger equation:

(7) $$\lambda^{2}{\left(dt\right)}^{\left[\frac{4}{f\left(\alpha \right)}\right]-2}\partial^{l}\partial_{l}\Psi +i\lambda {\left(dt\right)}^{\left[\frac{2}{f\left(\alpha \right)}\right]-1}\partial_{t}\Psi =0$$

Therefore, for the complex velocity fields (5), the dynamics of any polymeric biocomposite entities are described through Schrödinger type “regimes” at various scale resolutions (Schrödinger’s multifractal description).

Moreover, if it is chosen Ψ of the form (Madelung’s type choice):

(8) $$\Psi =\sqrt{\rho }e^{is},$$

where $\sqrt{\rho }\;$is the amplitude and s is the phase, then the multifractal complex velocity fields (5) take the explicit form:

(9) $${\hat{V}}^{i}=2\lambda {\left(dt\right)}^{\left[\frac{2}{f\left(\alpha \right)}\right]-1}\partial^{i}s-i\lambda {\left(dt\right)}^{\left[\frac{2}{f\left(\alpha \right)}\right]-1}\partial^{i}\ln\rho$$

which implies the real multifractal velocity fields:

(10) $$V_{D}^{i}=2\lambda {\left(dt\right)}^{\left[\frac{2}{f\left(\alpha \right)}\right]-1}\partial^{i}s$$

(11) $$V_{F}^{i}=\lambda {\left(dt\right)}^{\left[\frac{2}{f\left(\alpha \right)}\right]-1}\partial^{i}\ln\rho .$$

In (10), $V_{D}^{i}$ is the differential velocity field, while in (11), $V_{F}^{i}$ is the multifractal velocity field.

By (9), (10), and (11) and using the mathematical procedure from [64], the motion Equation (4) reduces to the multifractal Madelung equations:

(12) $$\partial_{t}V_{D}^{i}+V_{D}^{l}\partial_{l}V_{D}^{i}=-\partial^{i}Q$$

(13) $$\partial_{t}\rho +\partial_{l}\left(\rho V_{D}^{l}\right)=0$$

with  Q the multifractal specific potential:

(14) $$Q=-2\lambda^{2}{\left(dt\right)}^{\left[\frac{4}{f\left(\alpha \right)}\right]-2}\frac{\partial^{l}\partial_{l}\sqrt{\rho }}{\sqrt{\rho }}=-V_{F}^{i}V_{F}^{i}-\frac{1}{2}\lambda {\left(dt\right)}^{\left[\frac{2}{f\left(\alpha \right)}\right]-1}\partial_{l}V_{F}^{l}$$

The Equation (12) corresponds to the multifractal specific momentum conservation law, while Equation (13) corresponds to the multifractal states density conservation law. The multifractal specific potential (14) implies the multifractal specific force:

(15) $$F^{i}=-\partial^{i}Q=-2\lambda^{2}{\left(dt\right)}^{\left[\frac{4}{f\left(\alpha \right)}\right]-2}\partial^{i}\frac{\partial^{l}\partial_{l}\sqrt{\rho }}{\sqrt{\rho }}$$

which is a measure of the multifractality of the motion curves.

Therefore, for the multifractal complex velocity fields (9), the dynamics of any polymeric biocomposite are described through Madelung-type “regimes” at various scale resolution (Madelung’s multifractal description).

In this new context, any polymeric biocomposite entities are in a permanent interaction with a multifractal medium through the multifractal specific force (15); all polymeric biocomposites can be identified with a multifractal fluid, the dynamics of which are described by the multifractal Madelung’s equation (see (12)–(14)). The velocity field $V_{F}^{i}$ does not represent the contemporary dynamics. Since $V_{F}^{i}$ is missing from (13) this velocity field contributes to the transfer of the multifractal specific momentum and to the multifractal energy focus. Any analysis of *Q* should consider the “self” nature of the specific momentum transfer of multifractal type. Then, the conservation of the multifractal energy and the multifractal momentum that ensure the reversibility and the existence of the multifractal eigenstates.

If the multifractal tensor is considered:

(16) $${\hat{\tau }}^{il}=2\lambda^{2}{\left(dt\right)}^{\left[\frac{4}{f\left(\alpha \right)}\right]-2}\rho \partial^{i}\partial^{l}\ln\rho$$

the equation defining the multifractal forces that derive from the multifractal specific potential Q can be written in the form of an multifractal equilibrium equation:

(17) $$\rho \partial^{i}Q=\partial_{l}{\hat{\tau }}^{il}$$

Since ${\hat{\tau }}^{il}$ can be also written in the form:

(18) $${\hat{\tau }}^{il}=\eta \left(\partial_{l}V_{F}^{i}+\partial_{i}V_{F}^{l}\right)$$

with

(19) $$\eta =\lambda {\left(dt\right)}^{\left[\frac{2}{f\left(\alpha \right)}\right]-1}\rho$$

a multifractal linear constitutive equation for a multifractal “viscous fluid” can be highlighted. In such a context, the coefficient η can be interpreted as a multifractal dynamic viscosity coefficient of the multifractal fluid.

In the following, this tensor will be “made responsible” for the polymeric biocomposite behavior, when the material is subjected to tension and compression.

### 3.3. Stress–Strain Correlations in a Multifractal Paradigm

It is usually accepted the fact that stresses and strains at any point of any type of polymeric biocomposite, are represented, in a tensor form ($\sigma_{ik}$ for stress tensor of multifractal type and $\varepsilon_{ik}$ for the strain tensor of multifractal type, where i,k=1,2,3) with the characteristic cubic equation of multifractal type for strain having the form:

(20) $$\sigma^{3}-I_{1}\sigma^{2}+I_{2}\sigma -I_{3}=0$$

and the corresponding characteristic cubic equation of multifractal type for stress having the form:

(21) $$\varepsilon^{3}-J_{1}\varepsilon^{2}+J_{2}\varepsilon -J_{3}=0$$

In relations (20) and (21), $I_{1},I_{2},$ and $I_{3}$ are the invariants related to the stress tensor of multifractal type and $J_{1},J_{2}$, and $J_{3}$ are the invariants related to the strain tensor of multifractal type. If it is supposed that stresses of multifractal type and strains of multifractal type are linked to the same polymeric biocomposite point, then from a physical point of view, it is necessary to describe a relationship between these stresses and strains of multifractal type.

Ideally speaking, the best course of action is to find the most general relationship of multifractal type which, when referring to the case of tensors of multifractal type, should be invariant when considering orthogonal groups of multifractal type. In such a context, said relationship should establish a correlation between Is and Js of Equations (20) and (21).

As such, this general relationship of multifractal type can be written in the shape of a homographical transformation of multifractal type [64]:

(22) $$\varepsilon_{k}=\frac{\alpha \sigma_{k}+\beta }{\gamma \sigma_{k}+\delta },\;\;k=1,2,3$$

where $\sigma_{k}$ and $\varepsilon_{k}$ are the roots of Equations (20) and (21) and α,β,γ, and δ are real constants of multifractal type.

Thus, if the relation described by Equation (22) is taken to represent a constitutive law of multifractal type, then the parameters α,β,γ, and δ must be parameters of the polymeric biocomposite, of multifractal type. Using Equation (22) for a constitutive law of multifractal type, it is possible to describe the changes which occur in a polymeric biocomposite as a result of deformation using the material parameters of multifractal type α,β,γ, and δ.

As such, a unique state of stress of multifractal type related to a certain state of strain of multifractal type, even as the structure of the polymeric biocomposite is changing, can be described. This is in line with the usual definition for the stresses and strains, which do not depend on any polymeric biocomposite. Consequently, only the constitutive laws of multifractal type depend on the material.

Since every type of polymeric biocomposite changes its structure when the deformation process takes place, said change can be reflected in the fact that the matter coefficients α,β,γ, and δ, which make the constitutive law of multifractal type, vary with deformation. In these conditions, the matrix associated to the homographical transformation of multifractal type from (36)

(23) $$\hat{\alpha }=\left(\begin{array}{cc} \alpha & \beta \\ \gamma & \delta \end{array}\right)$$

becomes fundamental in the generation of the constitutive laws of multifractal type for any type of polymeric biocomposite—more precisely, in the differential geometry associated to this matrix.

In such a context, the “liquid wood” analysis in usual space, is reduced to obtain a relation between the matrix ensemble $\hat{\alpha }$ and an ensemble of σ values through which σ′ remains constant. Geometrically, this implies the searching of the ensemble of points (α, β, γ, δ), univocally corresponds to the values of the parameter σ. Using (22), the solution of the problem is reduced to a Riccati type gauge of multifractal type in the form:

(24) $$d\sigma +\omega_{1}\sigma^{2}+\omega_{2}\sigma +\omega_{3}=0$$

where the following notations are used [51,52]:

(25) $$\omega_{1}=\frac{\gamma d\alpha -\alpha d\gamma }{\Delta },\omega_{2}=\frac{\delta d\alpha -\alpha d\delta +\gamma d\beta -\beta d\gamma }{\Delta },\omega_{3}=\frac{\delta d\beta -\beta d\delta }{\Delta }$$

with

(26) Δ=αδ−γβ 

It is easy to verify the fact that the metric:

(27) $$ds^{2}=\frac{{\left(\delta d\alpha +\alpha d\delta -\gamma d\beta -\beta d\gamma \right)}^{2}}{4\Delta^{2}}-\frac{d\alpha d\delta -d\beta d\gamma }{\Delta }$$

is in a direct relation with the discriminant from of the quadratic polynomial (24)

(28) $$ds^{2}=\frac{1}{4}\left(\omega_{2}^{2}-4\omega_{1}\omega_{3}\right)$$

The three differentiable 1-forms from (25) completely define the coframe in every point of the absolute space. This coframe allows the translation of all geometric properties of the absolute space in the algebraic properties linked to (24) (evidently, all of them being of a multifractal type).

The simplest property refers to dynamics on the metric geodesics which can be correlated directly into statistical properties and from here the multi-fractalization through stochasticization. In this case, the 1-forms $\omega_{1},\;\omega_{2},\;\omega_{3}$ are differentiable in the same parameters, i.e.,

(29) $$\omega_{1}=a_{1}d\tau ,\;\;\omega_{2}=2a_{2}d\tau ,\;\;\omega_{3}=a_{3}d\tau$$

Along this geodesic, (24) becomes a Riccati type equation of multifractal type:

(30) $$\frac{d\sigma }{d\tau }=a_{1}\sigma^{2}+2a_{2}\sigma +a_{3}$$

$a_{1},a_{2},\;a_{3}$ being constants that characterize certains geodesics from the family.

The Equation (30) admits a direct integration, with three possible results:

(31) $$\sigma \left(\tau \right)=-\frac{a_{2}}{a_{1}}+\frac{\sqrt{\Delta }}{a_{1}}\tan\left[\sqrt{\Delta }\left(\tau -\tau_{0}\right)\right]\;for\;\Delta >0\;\sigma \left(\tau \right)=\frac{a\tau +b}{c\tau +d}\;for\;\Delta =0\;\sigma \left(\tau \right)=-\frac{a_{2}}{a_{1}}+\frac{\sqrt{\Delta }}{a_{1}}\coth\left[\sqrt{\Delta }\left(\tau -\tau_{0}\right)\right]\;for\;\Delta <0$$

Here, Δ and $\tau_{0}$ are defined by the relations:

(32) $$\Delta \equiv a_{1}a_{3}-a_{2}{}^{2},\;\;a_{2}=\sqrt{\Delta }\tan\left(\tau_{0}\sqrt{\Delta }\right)$$

For the first case (31) a,b,c, d are constants, not all of them arbitrary. Equation (31), depending on what the case may be, describes a deformation/compression process. It is noted that the main procedure can be applied for strain analysis as well.

**Figure 10:**
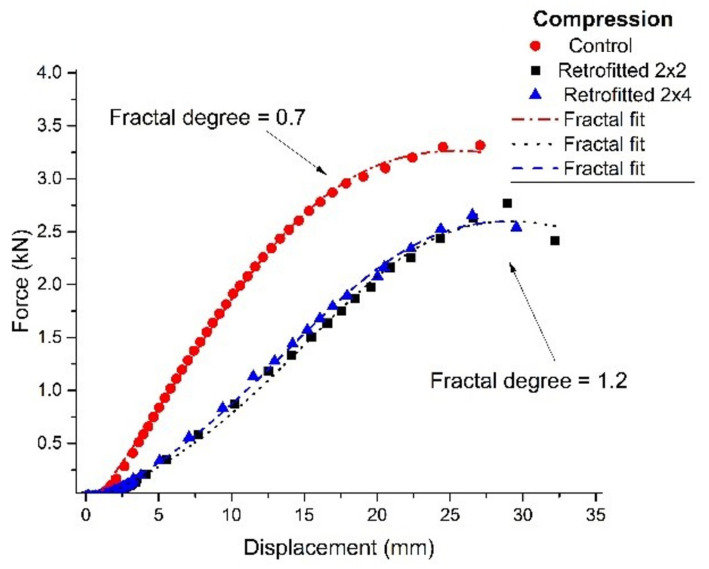
Behavior of samples rehabilitated with “liquid wood”, subjected to compression.

**Figure 11:**
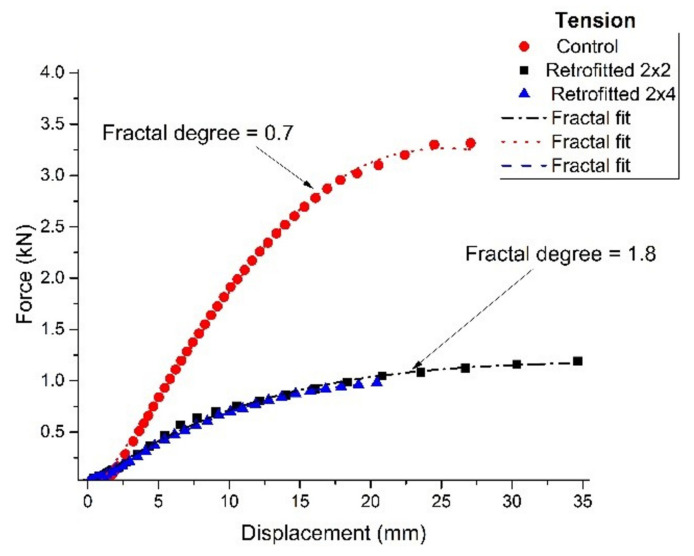
Behavior of samples rehabilitated with “liquid wood”, subjected to tension.

In Figure 10 and Figure 11, shown below, it is represented the behavior of samples rehabilitated with “liquid wood” subjected to compression (Figure 10) and tension (Figure 11). In order to fit the experimental data, the last form of Equation (31) was used, with the mention that all the constants were left “free”, $\frac{\sqrt{\Delta }}{a_{1}}$ being an approximation of the fractalization degree and τ was substituted with ε. In these conditions the last form of Equation (31) operates as a “true” constitutive law for “liquid wood”.

As it can be observed, there is a significant difference in fractalization between the control system and the “liquid wood” one. For tension a bigger mismatch can be noticed in the fractalization degree, which leads to a lower material performance. This is well in line with the understanding for the role of “liquid wood”, as it does not interact with timber, therefore, a set of fractal interactions that would be present at the contact surface is missing. This non-interaction scenario is the reasoning for the difference in the fractalization and performance mismatch as most of the behavior is an intrinsic one (only for the “liquid wood”). For compression, in the fractal paradigm presented here, most of the interactions are contained within the core of “liquid wood” with reduced interactions at the contact layer (see Figure 10), while for tension the main stress is induced through the contact layer reducing the performance and increasing the fractalization degree (see Figure 11).

## 4. Results and Discussion

Taking a closer look at the graphs, it is immediately noticeable that the samples behaved far better in compression than in tension. In particular, the performance in compression is very close to the control samples, with a value at breakage rated at approximately 2.7 kN, compared to approximately 3.3 kN for the control ones. This would equate to approximately 81% bearing capacity of the consolidated element, compared to the control one. As an observation, in all the tests performed in compression, “liquid wood” did not suffer any cracks or visible damage, the regular timber always failing first.

The performance in tension was lower than what was expected, with a value at breakage rated at approximately 1.2 kN for the retrofitted samples, compared to approximately 3.3 kN for the control ones. This would equate to approximately 36% bearing capacity of the consolidated element, compared to the control one. The obtained results indicate that “liquid wood” can be successfully used in elements where compression is to be expected. However, the results obtained in tension warrant further study and discussion.

One of the factors that determined the mediocre performance in tension can be attributed to the lack of interaction between the regular timber and “liquid wood”. It was noticed during material testing that “liquid wood” will not adhere in any meaningful way to timber, even though the two materials are of a polar nature and are biocompatible.

The missing adhesion does not seem to stem from improper processing of “liquid wood”, or of the wooden beams. “Liquid wood” is separately converted from a solid state (granules) into a flowing, “liquid” state, followed by thermo-injection and cooling. Similarly, the natural wood was kept and used in optimum laboratory conditions. Rather, there seems to be an interface issue with the two materials, which might be alleviated, for example, by the mixing of heat-activated adhesive ingredients in the “liquid wood” recipe.

Nevertheless, it was attempted to rectify this by using wooden screws, as a way of obtaining mechanical adhesion (and thus ensuring the transfer of loads) through the contact between timber, “liquid wood” and the screw flower head. In order to obtain better results in tension, additional steel pieces will have to be used, in order to facilitate the transfer of stresses between the natural wood and “liquid wood”. Although it was noticed that “liquid wood” will adhere to metal, this solution requires further investigations.

The better adherence to metal may be explained by previous experiments [77], which determined that lignin (present in “liquid wood”) will bind to metal because of a C–O link activation or through direct hydrogenolysis. In such a context, “liquid wood” may be used as a protection coating for metal surfaces, being inert with regard to acidic/base/UV actions.

Finally, it is difficult to compare the results presented in this paper with the ones presented in other manuscripts. The reason is that, to the knowledge of the authors, there are no other studies performed evaluating the performance of “liquid wood”, as a filler material, used for the repair of damaged wooden elements in civil engineering. There are, however, studies regarding mechanical characteristics of “liquid wood”, as a standalone material, in the form of thermo-injected samples subjected to tension and bending. More information regarding “liquid wood” mechanical testing can be found in [39,40,43,44].

## 5. Conclusions

The present paper is organized in two main parts: an experimental part and a theoretical part. As such, the following conclusions will be structured based on the organization of the paper.

Regarding the experimental aspects, the conclusions are:

(i) “Liquid wood” seems to be a good option as an eco-friendly, sustainable building material. Its properties appear to be well-suited to use in construction. At the current stage, the material cannot be applied in all situations, but the obtained results are encouraging.
(ii) It was decided to employ “liquid wood” in a rehabilitation scenario. Thus, tests both in tension and compression were made, which aim to identify the performance as well as the issues that the polymeric biocomposite might encounter in such situations. The obtained results were displayed in the graphs present at the end of Section 3 and briefly commented.
(iii) “Liquid wood” can be used as a repair material, in civil engineering. For compressed elements, like columns, “liquid wood” can be thermo-injected on spot, after proper groove preparation. It is fast to apply, cost-effective, and more environmentally friendly compared to other repair solutions. However, for elements subjected to tension, “liquid wood” requires additional load transfer steel pieces. Additional studies are required in order to determine the optimal solution for load transfer in tension. Once these issues are overcome, “liquid wood” might very well become a material of choice for the rehabilitation of all kinds of building elements.

Regarding the theoretical aspects, the conclusions are:

(i) It was decided to use Autodesk Inventor virtual simulation environment, in line with the expected employment of “liquid wood” as a thermo-injectable material into existing elements. A detailed simulation of a wooden beam which was rehabilitated with Arbofill trademarked product was performed, in a very similar scenario/setup to the one presented in the practical application. Furthermore, it was shown that such simulations can be performed for the other types of product as well. The simulation results confirm the data obtained from the experimental application;
(ii) The non-linear behaviors of “liquid wood” are described by means of a theoretical model in the fractal paradigm of motion. This model is based on the hypothesis that “liquid wood” can be assimilated, both structurally and functionally, to a multifractal object. Then, its entities are described through continuous, non-differentiable curves (multifractal curves);
(iii) The application of a covariance principle allows the obtainment of the conservation law of the specific momentum. From such a perspective, the description of the dynamics, both in a Schrödinger-type representation and in a hydrodynamic-type representation, at various scale resolutions, becomes operational;
(iv) In the hydrodynamic-type representation, at various scale resolutions, a stress-type tensor and its corresponding constitutive laws of multifractal type are presented. In the author’s opinion, this tensor becomes “responsible” for the behavior of “liquid wood” in tension and compression;
(v) Using several operational procedures (Riccati-type gauge, differential geometry in absolute space through homographic-type transformations etc.) will allow correlations between stresses and deformations;
(vi) The theoretical model was validated by means of data supplied by the experimental application.

## 6. Future Research Directions

One possible research direction is that the adhesion of “liquid wood” should definitely be studied in detail and methods for improving said adhesion should be explored. Otherwise, “liquid wood” will remain a material confined to a rather narrow palette of applications and uses.

Another possible research direction is that the structural properties of “liquid wood” should be further studied. If “liquid wood” should be used as a loadbearing material, then its properties concerning the behavior in tension, compression, shear etc., as well as in various combinations of efforts should be uncovered.

One more research direction is that improvement in injection technology (and its on-site employment) and groove preparation should be explored. It may be possible that with advances in both of these previously mentioned aspects, higher rehabilitation performance may be expected, such as better, more uniform groove coverage, and a better “liquid wood” paste layout.

## Data Availability

Not applicable.
